# 1*H*-Benzimidazol-3-ium-2-carboxyl­ate dihydrate

**DOI:** 10.1107/S1600536811017399

**Published:** 2011-05-14

**Authors:** Xing-Jun Yao, Qian Yuan

**Affiliations:** a College of Chemistry and Chemical Engineering, Liaocheng University, 252059 Liaocheng, Shandong, People’s Republic of China; b Guodian Liaocheng Power Co. Ltd, 252033 Liaocheng, Shandong, People’s Republic of China

## Abstract

The title compound, C_8_H_6_N_2_O_2_·2H_2_O, crystallized as a zwitterion with the carboxyl group deprotonated and the imidazole group protonated. The dihedral angle between the benzimidazole ring and the pendant –CO_2_ group is 0.62 (2)°. In the crystal, mol­ecules are linked into a three-dimensional network by N—H⋯O and O—H⋯O hydrogen bonds.

## Related literature

For the crystal structure of related zwitterionic benzimidazole-2-carb­oxy­lic acid monohydrate, see: Krawczyk *et al.* (2005 ▶). For the synthesis of the title compound, see: Thakurdesai *et al.* (2007 ▶).
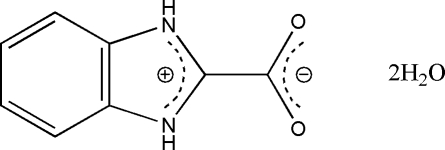


## Experimental

### 

#### Crystal data

C_8_H_6_N_2_O_2_·2H_2_O
*M* *_r_* = 198.18
Monoclinic, 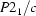

*a* = 6.8503 (15) Å
*b* = 7.3679 (17) Å
*c* = 18.939 (4) Å
β = 109.728 (7)°
*V* = 899.8 (3) Å^3^
*Z* = 4
Mo *K*α radiation
μ = 0.12 mm^−1^
*T* = 298 K
0.42 × 0.38 × 0.35 mm

#### Data collection

Bruker APEXII CCD diffractometer
Absorption correction: multi-scan (*SADABS*; Bruker, 2005 ▶) *T*_min_ = 0.952, *T*_max_ = 0.960
4496 measured reflections
1760 independent reflections
1342 reflections with *I* > 2σ(*I*)
*R*_int_ = 0.095

#### Refinement

*R*[*F* ^2^ > 2σ(*F* ^2^)] = 0.043
*wR*(*F* ^2^) = 0.111
*S* = 1.03
1760 reflections
128 parameters
H-atom parameters constrained
Δρ_max_ = 0.18 e Å^−3^
Δρ_min_ = −0.16 e Å^−3^

### 

Data collection: *APEX2* (Bruker, 2005 ▶); cell refinement: *SAINT* (Bruker, 2005 ▶); data reduction: *SAINT*; program(s) used to solve structure: *SHELXTL* (Sheldrick, 2008 ▶); program(s) used to refine structure: *SHELXTL*; molecular graphics: *SHELXTL*; software used to prepare material for publication: *SHELXTL*.

## Supplementary Material

Crystal structure: contains datablocks global, I. DOI: 10.1107/S1600536811017399/cv5086sup1.cif

Structure factors: contains datablocks I. DOI: 10.1107/S1600536811017399/cv5086Isup2.hkl

Supplementary material file. DOI: 10.1107/S1600536811017399/cv5086Isup3.cml

Additional supplementary materials:  crystallographic information; 3D view; checkCIF report

## Figures and Tables

**Table 1:** Hydrogen-bond geometry (Å, °)

*D*—H⋯*A*	*D*—H	H⋯*A*	*D*⋯*A*	*D*—H⋯*A*
O3—H6⋯O1	0.85	2.01	2.8608 (19)	174
N1—H1⋯O3^i^	0.86	1.86	2.7135 (18)	170
O4—H9⋯O3^i^	0.84	2.06	2.874 (2)	163
N2—H2*A*⋯O2^ii^	0.86	1.87	2.6708 (18)	155
O3—H7⋯O4^iii^	0.84	1.93	2.756 (2)	165
O4—H8⋯O1^iv^	0.85	2.53	3.142 (2)	130

Symmetry codes: (i) 
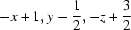
; (ii) 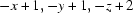; (iii) 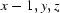; (iv) 
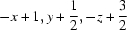
.

**Fig. 1.**
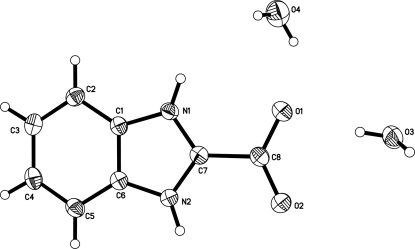
View of the title compound showing the atomic labeling and 30% probability displacement ellipsoids.

## supplementary crystallographic information

### Comment

Recently, the crystal structure of zwitterionic compound benzimidazole-2-carboxylic acid monohydrate (I) has been reported by Krawczyk *et al.* (2005). Herewith we present the crystal structure of its dihydrate form, the title compound (II).

In (II) (Fig. 1), the bond lengths and angles are normal and comparable with those observed in (I). The equal bond lengths of C7—N1 and C7—N2 of the imidazolium fragment, and C8—O1 and C8—O2 of the carboxylate group show both N atoms of the benzimidazole group are protonated and both O atoms of the carboxylate group are deprotonated. The dihedral angle between the benzimidazole and carboxylate groupe is 0.62 (2) °. The molecule is essentially planar, the r.m.s. deviation for all non-H atoms being 0.0249 Å. An extensive three-dimensional hydrogen-bonding network (Table 1) stabilize the crystal packing.

### Experimental

The title compound was synthesized according to the method reported in the literature (Thakurdesai *et al.*, 2007). Colourless single crystals suitable for X-ray diffraction were obtained by slow evaporation of an ethanol solution of the compound.

### Refinement

H atoms bonded to the water O atom were located in an electron density and refined with O—H distances constrained to 0.84-0.85 Å. Other H atoms were positioned geometrically and refined using a riding model, with C—H = 0.93 Å, and N—H = 0.86 Å. For those bound to C and N, *U*_iso_(H) = 1.2*U*_eq_(C, N), while for those bound to O, *U*_iso_(H) = 1.5 *U*_eq_(O).

### Crystal data

**Table:**

C_8_H_6_N_2_O_2_·2H_2_O	*F*(000) = 416
*M**_r_* = 198.18	*D*_x_ = 1.463 Mg m^−^^3^
Monoclinic, *P*2_1_/*c*	Mo *K*α radiation, λ = 0.71073 Å
Hall symbol: -P 2ybc	Cell parameters from 1540 reflections
*a* = 6.8503 (15) Å	θ = 2.3–26.2°
*b* = 7.3679 (17) Å	µ = 0.12 mm^−^^1^
*c* = 18.939 (4) Å	*T* = 298 K
β = 109.728 (7)°	Block, colorless
*V* = 899.8 (3) Å^3^	0.42 × 0.38 × 0.35 mm
*Z* = 4	

### Data collection

**Table:**

Bruker APEXII CCD diffractometer	1760 independent reflections
Radiation source: fine-focus sealed tube	1342 reflections with *I* > 2σ(*I*)
graphite	*R*_int_ = 0.095
φ and ω scans	θ_max_ = 26.0°, θ_min_ = 2.3°
Absorption correction: multi-scan (*SADABS*; Bruker, 2005)	*h* = −6→8
*T*_min_ = 0.952, *T*_max_ = 0.960	*k* = −9→9
4496 measured reflections	*l* = −23→17

### Refinement

**Table:**

Refinement on *F*^2^	Secondary atom site location: difference Fourier map
Least-squares matrix: full	Hydrogen site location: inferred from neighbouring sites
*R*[*F*^2^ > 2σ(*F*^2^)] = 0.043	H-atom parameters constrained
*wR*(*F*^2^) = 0.111	*w* = 1/[σ^2^(*F*_o_^2^) + (0.041*P*)^2^ + 0.0001*P*] where *P* = (*F*_o_^2^ + 2*F*_c_^2^)/3
*S* = 1.03	(Δ/σ)_max_ = 0.001
1760 reflections	Δρ_max_ = 0.18 e Å^−^^3^
128 parameters	Δρ_min_ = −0.16 e Å^−^^3^
0 restraints	Extinction correction: *SHELXTL* (Sheldrick, 2008), Fc^*^=kFc[1+0.001xFc^2^λ^3^/sin(2θ)]^-1/4^
Primary atom site location: structure-invariant direct methods	Extinction coefficient: 0.097 (8)

### Special details

**Table:**

Geometry. All e.s.d.'s (except the e.s.d. in the dihedral angle between two l.s. planes)are estimated using the full covariance matrix. The cell e.s.d.'s are takeninto account individually in the estimation of e.s.d.'s in distances, anglesand torsion angles; correlations between e.s.d.'s in cell parameters are onlyused when they are defined by crystal symmetry. An approximate (isotropic)treatment of cell e.s.d.'s is used for estimating e.s.d.'s involving l.s. planes.
Refinement. Refinement of *F*^2^ against ALL reflections. The weighted *R*-factor *wR* and goodness of fit *S* are based on *F*^2^, conventional *R*-factors *R* are based on *F*, with *F* set to zero for negative *F*^2^. The threshold expression of *F*^2^ > σ(*F*^2^) is used only for calculating *R*-factors(gt) *etc*. and is not relevant to the choice of reflections for refinement. *R*-factors based on *F*^2^ are statistically about twice as large as those based on *F*, and *R*- factors based on ALL data will be even larger.

### Fractional atomic coordinates and isotropic or equivalent isotropic displacement parameters (Å^2^)

**Table:**

	*x*	*y*	*z*	*U*_iso_*/*U*_eq_	
O1	0.4666 (2)	0.4099 (2)	0.80063 (6)	0.0549 (4)	
O2	0.36643 (18)	0.48692 (18)	0.89664 (6)	0.0503 (4)	
N1	0.8395 (2)	0.27481 (18)	0.90210 (7)	0.0352 (4)	
H1	0.8355	0.2552	0.8569	0.042*	
N2	0.74817 (19)	0.35912 (18)	0.99579 (7)	0.0354 (4)	
H2A	0.6754	0.4029	1.0209	0.043*	
C8	0.4882 (3)	0.4223 (2)	0.86784 (9)	0.0378 (4)	
C7	0.6902 (3)	0.3506 (2)	0.92144 (8)	0.0346 (4)	
C1	1.0037 (2)	0.2319 (2)	0.96623 (9)	0.0338 (4)	
C2	1.1954 (3)	0.1538 (2)	0.97674 (9)	0.0392 (4)	
H2	1.2351	0.1172	0.9366	0.047*	
C3	1.3236 (3)	0.1332 (2)	1.04979 (10)	0.0443 (5)	
H3	1.4546	0.0830	1.0594	0.053*	
C4	1.2623 (3)	0.1857 (3)	1.11020 (10)	0.0451 (5)	
H4	1.3532	0.1678	1.1588	0.054*	
C5	1.0734 (3)	0.2625 (2)	1.10005 (9)	0.0415 (5)	
H5	1.0331	0.2970	1.1403	0.050*	
C6	0.9443 (2)	0.2864 (2)	1.02604 (9)	0.0344 (4)	
O3	0.1521 (2)	0.67445 (19)	0.73470 (6)	0.0542 (4)	
H7	0.0403	0.6248	0.7338	0.081*	
H6	0.2413	0.5942	0.7569	0.081*	
O4	0.7592 (2)	0.5359 (2)	0.70601 (8)	0.0747 (5)	
H8	0.6747	0.5904	0.7228	0.112*	
H9	0.7617	0.4321	0.7248	0.112*	

### Atomic displacement parameters (Å^2^)

**Table:**

	*U*^11^	*U*^22^	*U*^33^	*U*^12^	*U*^13^	*U*^23^
O1	0.0504 (8)	0.0778 (10)	0.0330 (7)	0.0132 (7)	0.0096 (6)	−0.0016 (6)
O2	0.0422 (7)	0.0669 (9)	0.0417 (8)	0.0109 (7)	0.0138 (6)	−0.0024 (6)
N1	0.0398 (8)	0.0360 (8)	0.0295 (7)	0.0008 (6)	0.0114 (6)	−0.0015 (6)
N2	0.0344 (8)	0.0386 (9)	0.0340 (8)	0.0029 (6)	0.0126 (6)	−0.0001 (6)
C8	0.0391 (10)	0.0389 (10)	0.0336 (9)	−0.0026 (8)	0.0099 (7)	−0.0008 (7)
C7	0.0379 (9)	0.0324 (9)	0.0334 (9)	−0.0022 (7)	0.0118 (7)	−0.0006 (7)
C1	0.0357 (9)	0.0299 (9)	0.0341 (9)	−0.0029 (7)	0.0097 (7)	0.0013 (7)
C2	0.0407 (10)	0.0342 (9)	0.0446 (10)	−0.0003 (8)	0.0169 (8)	−0.0006 (7)
C3	0.0390 (10)	0.0387 (11)	0.0523 (11)	0.0026 (8)	0.0114 (8)	0.0028 (8)
C4	0.0430 (11)	0.0422 (11)	0.0411 (10)	−0.0019 (8)	0.0025 (8)	0.0042 (8)
C5	0.0471 (11)	0.0415 (10)	0.0338 (9)	−0.0015 (8)	0.0110 (7)	−0.0009 (7)
C6	0.0354 (9)	0.0314 (9)	0.0350 (9)	−0.0036 (7)	0.0101 (7)	−0.0001 (7)
O3	0.0540 (8)	0.0618 (9)	0.0446 (8)	0.0048 (7)	0.0137 (6)	0.0122 (6)
O4	0.0629 (10)	0.0848 (12)	0.0758 (11)	−0.0023 (8)	0.0225 (8)	0.0131 (8)

### Geometric parameters (Å, °)

**Table:**

O1—C8	1.234 (2)	C2—H2	0.9300
O2—C8	1.236 (2)	C3—C4	1.400 (3)
N1—C7	1.321 (2)	C3—H3	0.9300
N1—C1	1.385 (2)	C4—C5	1.366 (2)
N1—H1	0.8600	C4—H4	0.9300
N2—C7	1.3291 (18)	C5—C6	1.393 (2)
N2—C6	1.379 (2)	C5—H5	0.9300
N2—H2A	0.8601	O3—H7	0.8435
C8—C7	1.508 (2)	O3—H6	0.8525
C1—C2	1.385 (2)	O4—H8	0.8496
C1—C6	1.386 (2)	O4—H9	0.8415
C2—C3	1.374 (2)		
			
C7—N1—C1	109.21 (13)	C3—C2—H2	121.8
C7—N1—H1	125.4	C1—C2—H2	121.8
C1—N1—H1	125.4	C2—C3—C4	121.77 (17)
C7—N2—C6	108.83 (13)	C2—C3—H3	119.1
C7—N2—H2A	125.6	C4—C3—H3	119.1
C6—N2—H2A	125.5	C5—C4—C3	122.08 (16)
O1—C8—O2	128.31 (16)	C5—C4—H4	119.0
O1—C8—C7	115.57 (15)	C3—C4—H4	119.0
O2—C8—C7	116.11 (14)	C4—C5—C6	116.33 (16)
N1—C7—N2	109.33 (13)	C4—C5—H5	121.8
N1—C7—C8	125.56 (14)	C6—C5—H5	121.8
N2—C7—C8	125.09 (14)	N2—C6—C1	106.67 (13)
N1—C1—C2	132.13 (15)	N2—C6—C5	131.73 (15)
N1—C1—C6	105.95 (14)	C1—C6—C5	121.59 (15)
C2—C1—C6	121.92 (15)	H7—O3—H6	101.8
C3—C2—C1	116.30 (16)	H8—O4—H9	100.9
			
C1—N1—C7—N2	−0.27 (19)	C1—C2—C3—C4	1.0 (3)
C1—N1—C7—C8	177.85 (15)	C2—C3—C4—C5	−0.9 (3)
C6—N2—C7—N1	0.32 (18)	C3—C4—C5—C6	−0.1 (3)
C6—N2—C7—C8	−177.81 (16)	C7—N2—C6—C1	−0.25 (17)
O1—C8—C7—N1	−1.3 (3)	C7—N2—C6—C5	−179.71 (17)
O2—C8—C7—N1	179.22 (16)	N1—C1—C6—N2	0.09 (17)
O1—C8—C7—N2	176.57 (16)	C2—C1—C6—N2	179.40 (15)
O2—C8—C7—N2	−2.9 (3)	N1—C1—C6—C5	179.61 (15)
C7—N1—C1—C2	−179.11 (17)	C2—C1—C6—C5	−1.1 (2)
C7—N1—C1—C6	0.11 (18)	C4—C5—C6—N2	−179.49 (17)
N1—C1—C2—C3	179.09 (17)	C4—C5—C6—C1	1.1 (2)
C6—C1—C2—C3	0.0 (2)		

### Hydrogen-bond geometry (Å, °)

**Table:**

*D*—H···*A*	*D*—H	H···*A*	*D*···*A*	*D*—H···*A*
O3—H6···O1	0.85	2.01	2.8608 (19)	174
N1—H1···O3^i^	0.86	1.86	2.7135 (18)	170
O4—H9···O3^i^	0.84	2.06	2.874 (2)	163
N2—H2A···O2^ii^	0.86	1.87	2.6708 (18)	155
O3—H7···O4^iii^	0.84	1.93	2.756 (2)	165
O4—H8···O1^iv^	0.85	2.53	3.142 (2)	130

Symmetry codes: (i) −*x*+1, *y*−1/2, −*z*+3/2; (ii) −*x*+1, −*y*+1, −*z*+2; (iii) *x*−1, *y*, *z*; (iv) −*x*+1, *y*+1/2, −*z*+3/2.

## References

[bb1] Bruker (2005). *APEX2*, *SAINT* and *SADABS* Bruker AXS Inc., Madison, Wisconsin, USA.

[bb2] Krawczyk, S., Gdaniec, M. & Sa˛czewski, F. (2005). *Acta Cryst.* E**61**, o4185–o4187.

[bb3] Sheldrick, G. M. (2008). *Acta Cryst.* A**64**, 112–122. 10.1107/S0108767307043930 18156677

[bb4] Thakurdesai, P. A., Wadodkar, S. G. & Chopade, C. T. (2007). *Pharmacology­online*, **1**, 314–329.

